# Generation and Immunogenicity of a Recombinant Pseudorabies Virus Co-Expressing Classical Swine Fever Virus E2 Protein and Porcine Circovirus Type 2 Capsid Protein Based on Fosmid Library Platform

**DOI:** 10.3390/pathogens8040279

**Published:** 2019-12-01

**Authors:** Muhammad Abid, Teshale Teklue, Yongfeng Li, Hongxia Wu, Tao Wang, Hua-Ji Qiu, Yuan Sun

**Affiliations:** State Key Laboratory of Veterinary Biotechnology, Harbin Veterinary Research Institute, Chinese Academy of Agricultural Sciences, Harbin 150069, China; 2017y90100057@caas.cn (M.A.); 2017y90100180@caas.cn (T.T.); liyongfeng@caas.cn (Y.L.); wuhongxia@caas.cn (H.W.); wangtao02@caas.cn (T.W.)

**Keywords:** pseudorabies virus, classical swine fever virus, porcine circovirus type 2, E2 and Cap, fosmid

## Abstract

Pseudorabies (PR), classical swine fever (CSF), and porcine circovirus type 2 (PCV2)-associated disease (PCVAD) are economically important infectious diseases of pigs. Co-infections of these diseases often occur in the field, posing significant threat to the swine industry worldwide. gE/gI/TK-gene-deleted vaccines are safe and capable of providing full protection against PR. Classical swine fever virus (CSFV) E2 glycoprotein is mainly used in the development of CSF vaccines. PCV2 capsid (Cap) protein is the major antigen targeted for developing PCV2 subunit vaccines. Multivalent vaccines, and especially virus-vectored vaccines expressing foreign proteins, are attractive strategies to fight co-infections for various swine diseases. The gene-deleted pseudorabies virus (PRV) can be used to develop promising and economical multivalent live virus-vectored vaccines. Herein, we constructed a gE/gI/TK-gene-deleted PRV co-expressing E2 of CSFV and Cap of PCV2 by fosmid library platform established for PRV, and the expression of E2 and Cap proteins was confirmed using immunofluorescence assay and western blotting. The recombinant virus propagated in porcine kidney 15 (PK-15) cells for 20 passages was genetically stable. The evaluation results in rabbits and pigs demonstrate that rPRVTJ-delgE/gI/TK-E2-Cap elicited detectable anti-PRV antibodies, but not anti-PCV2 or anti-CSFV antibodies. These findings provide insights that rPRVTJ-delgE/gI/TK-E2-Cap needs to be optimally engineered as a promising trivalent vaccine candidate against PRV, PCV2 and CSFV co-infections in future.

## 1. Introduction

Pseudorabies (PR), or Aujeszky’s disease (AD), is a lethal viral disease of pigs, and its causative agent is pseudorabies virus (PRV) [1]. PRV (also classified as suid herpesvirus 1) belongs to the *Varicellovirus* genus *Alphaherpesvirinae* subfamily within the *Herpesviridae* family. The genome of PRV is a double-stranded linear DNA molecule about 143 kb in size [2], and comprises almost seventy open reading frames (ORFs) encoding for at least 70–100 viral proteins, which include structural and non-structural proteins, replicases, and virulence-associated proteins [3]. PR is a very devastating disease manifested by higher fatality in neonates, respiratory distress in fattening pigs, and abortions and stillbirths in pregnant sows. Glycoprotein E (gE)-deleted PR vaccines are safe and efficacious and have been widely applied in several countries to control and eliminate PR [4]. As with other herpesviruses which have been used as a vector to develop recombinant viruses, PRV also contains many non-essential genes, and the gene-deleted PRV strains could be utilized as a vaccine vector in which various foreign genes are inserted and stably expressed for developing multivalent vaccines against PR and other major diseases of swine [5]. Previously, our studies showed that PRV variant based gE/gI- and gE/gI/TK-gene-deleted mutants were highly safe and efficacious in vulnerable animals and completely protected the immunized animals challenged by the lethal PRV Tianjin (PRV TJ) strain [6,7], demonstrating that the gene-deleted PRV could be used as a potential vector to manufacture virus-vectored vaccines.

Classical swine fever (CSF) is an economically significant viral disease of swine distributed worldwide [8]. It is a multisystemic, highly transmissible disease, resulting in hemorrhages, fever, immunosuppression and ataxia leading to huge economic losses [9]. CSF is caused by classical swine fever virus (CSFV), which belongs to the genus *Pestivirus,* family *Flaviviridae* of the viruses [10]. The CSFV genome comprises a single large ORF coding for a polyprotein subsequently processed into 4 structural proteins (C, E^rns^, E1, E2) and 8 non-structural (N^pro^, p7, NS2, NS3, NS4A, NS4B, NS5A and NS5B) [11]. The E2 glycoprotein of CSFV is capable of eliciting protective immune response against CSF in pigs, and the genetically engineered CSF vaccines are widely based on it [12,13]. Presently, vaccination is an important strategy in many countries for the control and eradication of CSF [14,15]. C-strain vaccine and modified live vaccines (MLVs) are significantly important because they play a critical role in controlling CSF, but these vaccines don’t possess the property of differentiation of infected from vaccinated animals (DIVA) [16]. Therefore, it is desirable to develop alternative vaccine strategies such as subunit or virus-vectored vaccines with higher efficacy and the DIVA property.

Porcine circovirus type 2 (PCV2) is the major causative agent of post-weaning multi-systemic wasting syndrome (PMWS), a multifactorial disease mostly affecting nursery and growing pigs [17]. Together with PWMS, PCV2 is responsible for several disease symptoms and syndromes generally regarded as PCV2-associated disease (PCVAD), which is the leading cause of huge monetary losses to the swine industry across the world [18]. Additionally, PCV2 also co-infects with other pathogens of swine for example PRV, CSFV, porcine parvovirus (PPV) and porcine reproductive and respiratory syndrome (PRRSV), resulting in more severe wasting disease [19,20]. PCV2 is a non-enveloped, smallest known DNA virus belonging to the genus *Circovirus,* within family *Circoviridae*. The genome is a single-stranded, circular DNA of approximately 1.7 kb in size encoding two major ORFs, that is, the ORF1 (*rep* gene), encoding for Rep proteins which is critical for virus replication, and the ORF2 (*cap* gene) encoding for capsid (Cap) protein solely responsible for virus capsid formation [21]. Vaccines are the primary strategy to fight PCV2 infections. Cap is the only structural protein and is the main antigen targeted for PCV2 vaccines and diagnostics development [22]. Cap protein can be expressed in *Escherichia coli* or baculovirus, resulting in the formation of virus-like particles (VLPs) [23]. Presently, the commercially available subunit vaccines against PCV2 are manufactured by expressing Cap protein in the baculovirus expression system, and are very effective in controlling PCV2 infections, reducing viremia and elimination of PCVAD [24,25]. However, the cost of vaccination for PCV2 remains a big concern for the majority of pork producers [26].

Recently licensed commercial PCV2 vaccines are highly effective and capable of eliciting protective immune responses in vaccinated pigs [27,28], but this is not sterilizing immunity and pigs remain susceptible to infection by drifted or novel PCV2 strains [29]. PCV2 has a relatively high mutation rate (1.2 × 10^−3^ substitutions per year), similar to that of RNA viruses [30]. Imperfect vaccine programs combined with this inherent high mutation rate may lead to the evolution of novel PCV2 strains that are not protected by the current PCV2 vaccines [28,31]. For example, it has been demonstrated that mutations in Cap gene are responsible for the evolution of vaccination-escape PCV2 mutants [32]. Therefore, it is desirable to develop novel vaccines based on currently circulating PCV2 genotypes and/or strains. Also, the PCV2 multivalent vaccines with other co-infecting pathogens of swine could simplify the vaccination programs and minimize the cost. 

PRV, PCV2, and CSFV infections, as mentioned above, can be controlled by vaccination. However, there is a need to develop a trivalent vaccine candidate for these economically important pathogens of pigs that could reduce the cost of vaccines and simplify the vaccination program. Hence, the purpose of the present study was to generate a recombinant PRV (rPRVTJ-delgE/gI/TK-E2-Cap) co-expressing the E2 protein of CSFV and Cap protein of PCV2 by using fosmid library platform, based on a gE/gI/TK gene-deficient PRV TJ backbone. The recombinant virus rPRVTJ-delgE/gI/TK-E2-Cap was generated, its stability and foreign gene expression were analyzed *in vitro*, and its safety and immunogenicity experiments were performed in rabbits and pigs.

## 2. Results

### 2.1. Rescue and Characterization of rPRVTJ-delgE/gI/TK-E2-Cap

**Figure 1:**
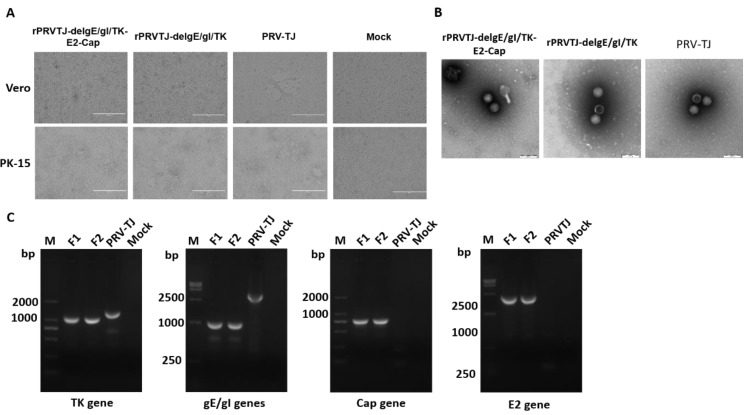
Rescue and characterization of the recombinant virus rPRVTJ-delgE/gI/TK-E2-Cap. **A.** Cytopathic effects (CPEs) observed on Vero or porcine kidney 15 (PK-15) cells. Vero cells were transfected with different combinations fosmids, positive control (parental virus PRV TJ strain) and negative control (missing one fosmid). At 72-96 hpt, CPEs were visualized and supernatants were collected. PK-15 cells were infected with the collected supernatants and the CPEs were visualized at 24 hpi. **B.** Electron microscopy. Transmission electron microscopy of the rPRVTJ-delgE/gI/TK-E2-Cap and rPRVTJ-delgE/gI/TK viral particles, the parental virus PRVTJ was used as positive control. Scale bars are presented. **C.** PCR analysis of deleted/inserted genes. PCR analysis of deleted thymidine kinase (TK), gE/gI genes, and E2 and Cap genes inserted in the DNA of rPRVTJ-delgE/gI/TK-E2-Cap using specific primers targeting TK, gE/gI, US9 (E2 gene) and US4 (Cap gene) regions, respectively. DNA of PRVTJ was used as the negative control and non-DNA templates were used as mock.

The concentration of each extracted fosmid used in the study was adjusted according to 2 μg. Each group contained five overlapping fosmids (the modified fosmids, fosmid-f-ΔTK, fosmid-s-ΔgE/gI, and fosmid-s-ΔgE/gI-US9E2-US4Cap and other fosmids that cover the entire genome), and the transfection mixture was prepared by mixing 2 μg of each fosmid with 30 μL of X-treme GENE HP DNA transfection reagent in 1 mL of Dulbecco’s modified Eagle’s medium (DMEM) and incubated for 20 min at room temperature. Subsequently, the transfection mixture was used to transfect Vero cells for virus rescue. The typical PRV Cytopathic effects (CPEs) were visualized in Vero cells at 72–96 h post-transfection (hpt) confirming that recombinant virus had been successfully rescued. The CPEs were also observed on porcine kidney 15 (PK-15) cells infected with rPRVTJ**-**delgE/gI/TK-E2-Cap, at 24 h post-infection (hpi) (Figure 1A). When examined using an electron microscope, the rPRVTJ-delgE/gI/TK-E2-Cap and rPRVTJ-delgE/gI/TK particles were morphologically similar to that of parental PRVTJ strain with an apparent external envelope (Figure 1B). To verify TK/gE/gI deletion and E2/Cap gene insertion at the target positions, viral DNA were extracted and analyzed by PCR (Figure 1C) and sequencing.

### 2.2. Confirmation of E2 and Cap Expression

**Figure 2:**
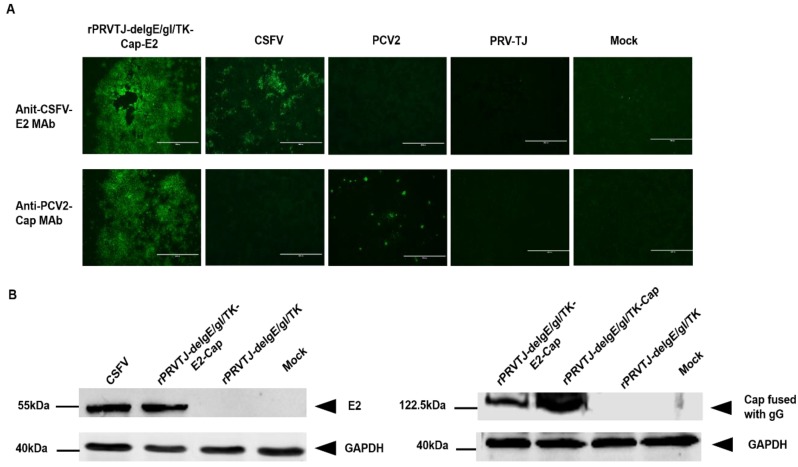
Confirmation of E2 and Cap expression. **A.** Confirmation of E2 and Cap expression by immunofluorescence assay (IFA). The E2 and Cap proteins expression was verified by IFA in PK-15 cells infected with rPRVTJ-delgE/gI/TK-E2-Cap. Briefly, PK-15 cells were infected with classical swine fever virus (CSFV), porcine circovirus type 2 (PCV2) rPRVTJ-delgE/gI/TK-E2-Cap and PRV TJ at a multiplication of infectivity (MOI) of 1 or left un-infected (mock). At 48 hpi, the cells were fixed and analyzed by IFA using the anti-E2 MAb HQ06 and anti-Cap MAb 36A9 primary antibodies and fluorescein isothiocyanate (FITC)-labeled anti-mouse secondary antibody. Bars, 400 nm. The results were visualized under a fluorescent microscope (TE2000-U Nikon, Japan). **B.** Expression of E2 and Cap by Western blotting. To confirm E2 and Cap expression, PK-15 cells were either infected with the CSFV, rPRVTJ-delgE/gI/TK-E2-Cap, rPRVTJdelgE/gI/TK-Cap, rPRVTJ-delgE/gI/TK or mock infected. At 48 hpi, the cells were lysed and assayed by Western blotting using the anti-E2 MAb HQ06 and anti-Cap MAb 36A9 (1:300) in PBS, subsequently incubated with IRDye 800CW-labeled goat anti-mouse secondary antibody (Li-Cor). GAPDH was used as a loading control. The arrows indicate the representative bands of the CSFV E2 protein and PCV2 Cap fused with the PRV gG.

The E2 and Cap expression in rPRVTJ-delgE/gI/TK-E2-Cap was first confirmed by using immunofluorescence assay (IFA) in infected PK-15 cells. The E2 and Cap proteins expression was evidenced by distinct fluorescence in the cells infected with rPRVTJ-delgE/gI/TK-E2-Cap, however, no fluorescence was detectable in cells infected with PRVTJ or mock cells (Figure 2A). Western blot tests of rPRVTJ-delgE/gI/TK-E2-Cap-infected PK-15 cell lysate using anti-E2 and anti-Cap MAbs revealed distinct bands of E2 and Cap-fused with gG were about 55 and 122.5 kDa, respectively (Figure 2B). In both IFA and Western blotting, it was confirmed that E2 and Cap were expressed by rPRVTJ-delgE/gI/TK-E2-Cap.

### 2.3. Growth Kinetics and Stability of Recombinant Viruses

**Figure 3:**
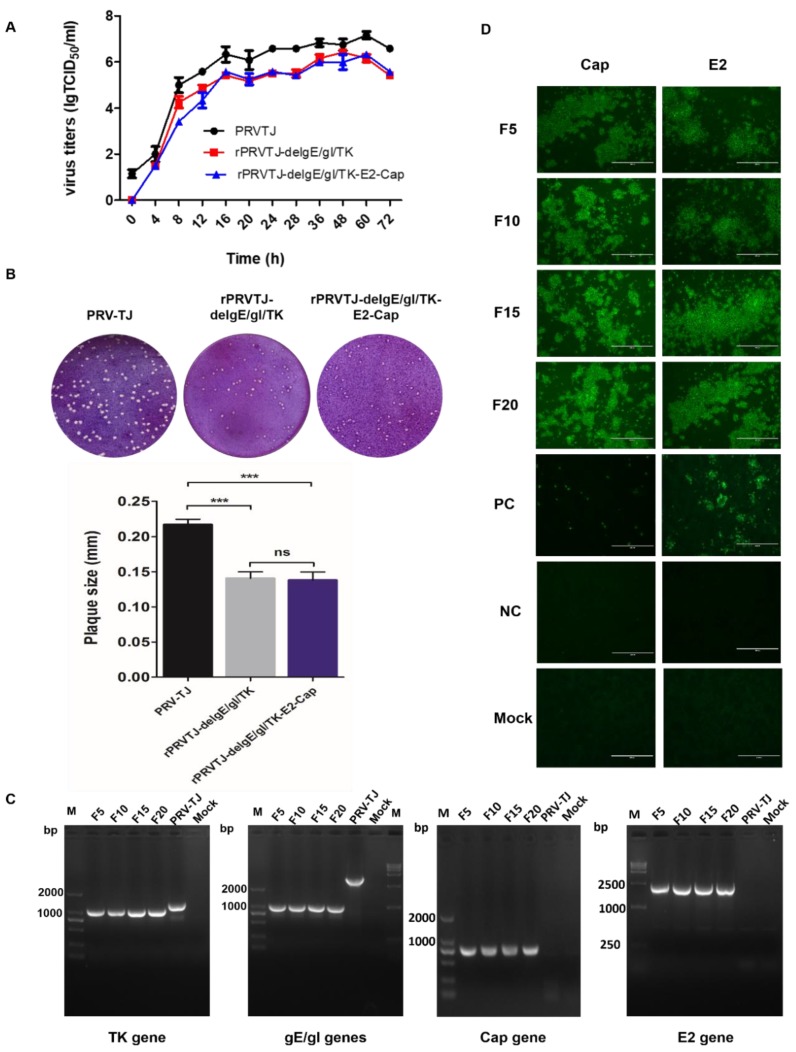
The replication kinetics and stability of recombinant viruses**. A.** One-step growth curve. PK-15 cells were infected with the rPRVTJ-delgE/gI/TK-E2-Cap, PRVTJ-delgE/gI/TK or PRVTJ at an MOI of 10. The cell culture supernatants were harvested at various intervals and used to determine viral titers. **B.** Plaque assay. The rPRVTJ-delgE/gI/TK-E2-Cap, rPRVTJ-delgE/gI/TK and PRVTJ were diluted 10-fold by serial dilution to achieve a titer required to form single plaques. The experiments were conducted in three repeats, and the corresponding results are presented. **C.** PCR identification. The recombinant virus propagated in PK-15 cells for 20 passages. The genome of the rPRVTJ-delgE/gI/TK-E2-Cap (F5, F10, F15 and F20) as a template for the amplification of ΔTK, ΔgE/gI, E2 and Cap genes by PCR. The PRVTJ genome was used as a negative control and non-DNA templates were used as mock. **D.** E2 and Cap expression by IFA. Expression of E2 and Cap proteins in PK-15 cells infected with the recombinant virus rPRVTJ-delgE/gI/TK-E2-Cap (F5, F10, F15 and F20) was confirmed by IFA using anti-E2 MAb HQ06 and anti-Cap MAb 36A9 as primary antibodies and FITC-labeled anti-mouse immunoglobulin G (IgG)as secondary antibody. Bars, 400 nm.

The growth kinetics and magnitude of the recombinant virus rPRVTJ-delgE/gI/TK-E2-Cap were identical to that of the rPRVTJ-delgE/gI/TK, indicating that E2 and Cap insertion in the PRV genome did not affect the replication of rPRVTJ-delgE/gI/TK-E2-Cap (Figure 3A). The plaque morphology of rPRVTJ-delgE/gI/TK-E2-Cap and rPRVTJ-delgE/gI/TK were quite similar, but it was slightly different from the PRV TJ strain (Figure 3B). PCR amplification and sequencing of deleted TK and gE/gI genes and, E2 and Cap genes inserted in the rPRVTJ-delgE/gI/TK-E2-Cap passaged for 20 generations in PK-15 cells were performed and it was confirmed that the generated recombinant PRV was genetically stable without any genetic alteration (Figure 3C). E2 and Cap expression at different passages of the recombinant virus was also confirmed by IFA with anti-E2 (HQ06) and anti-Cap (36A9) MAbs, respectively (Figure 3D).

### 2.4. Safety of rPRVTJ-delgE/gI/TK-E2-Cap for Rabbits

**Figure 4:**
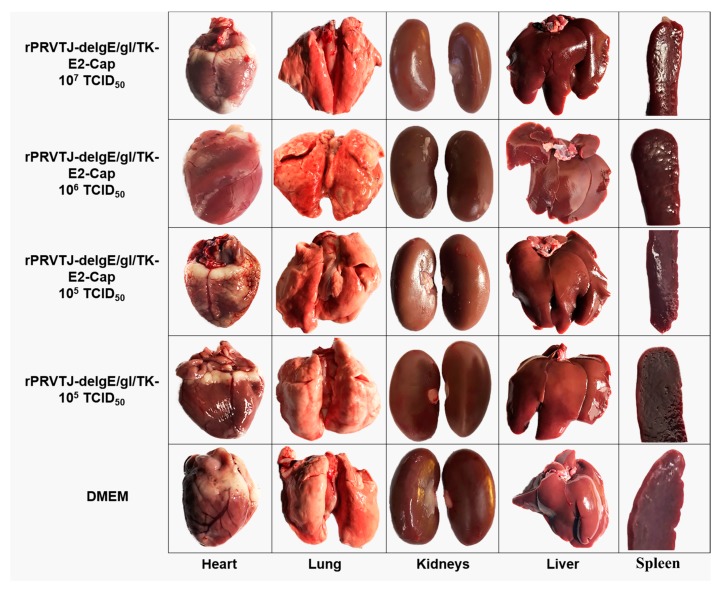
Gross pathological examination of various organs of immunized rabbits. Groups of rabbits (n = 5) were vaccinated with different doses (10*^7^*, 10*^6^* and 10*^5^* TCID_50_) of rPRVTJ-delgE/gI/TK-E2-Cap, 10*^5^* TCID_50_ of rPRVTJ-delgE/gI/TK, or DMEM. At six weeks post-immunization, all the immunized rabbits were euthanized and various organs, that is, lungs, liver, kidneys, heart and spleen were collected for gross pathological examination.

All of the rabbits immunized with different doses (10^7^, 10^6^ and 10^5^ TCID_50_) of rPRVTJ-delgE/gI/TK-E2-Cap did not exhibit any PRV specific clinical signs such as fever etc. Additionally, there were no pathological changes (Figure 4) and/or no PRV DNA was detected in the various organs collected from all of the immunized rabbits.

### 2.5. Immunogenicity of rPRVTJ-delgE/gI/TK-E2-Cap in Rabbits and Pigs

The PRV gB-specific antibodies were determined in the sera of rabbits immunized with rPRVTJ-delgE/gI/TK (10^7^ TCID_50_) at day 7 post-immunization, and all the rabbits immunized with (10^6^ TCID_50_, 10^5^ TCID_50_ of rPRVTJ-delgE/gI/TK-E2 developed gB-specific antibodies at day 14 post-immunization **(**Table 1**)**. The gE-specific antibodies were not detectable in all the rabbits before and after immunization confirming the deletion of the gE gene. 

The PRV gB-specific antibodies were determined in the sera of pigs immunized with 10^6^ TCID_50_ rPRVTJ-delgE/gI/TK-E2-Cap or rPRVTJ-delgE/gI/TK at day 7 post-immunization. The pigs vaccinated with 10^6^ TCID_50_ of rPRVTJ-delgE/gI/TK-E2-Cap developed almost similar levels of gB-specific antibodies to those immunized with 10^6^ TCID_50_ rPRVTJ-delgE/gI/TK **(**Table 2**)**. There were no gE-specific antibodies in the sera of all the pigs immunized with rPRVTJ-delgE/gI/TK-E2-Cap or rPRVTJ-delgE/gI/TK. Blocking ELISA results for CSFV E2 and PCV2 Cap showed that rPRVTJ-delgE/gI/TK-E2-Cap did not induce detectable anti-PCV2 or anti-CSFV antibodies.

## 3. Discussion

Despite significant efforts towards the control and eradication of PR and CSF, these diseases are still endemic in swine in many countries [33]. It has been reported that co-infections of PRV, CSFV, PCV2 or other viruses such as PPV and PRRSV often occur in the swine populations [34,35,36], leading to more severe wasting diseases. Therefore, the development of a multivalent vaccine would be of great significance to address co-infections, simplify immunization programs, and reduce the cost associated with infection by these pathogens of swine.

The genome of PRV is very long and contains unique long and unique short regions (UL and US), large inverted repeats with the US region flanked by internal repeats (IRs), and terminal repeats (TRs) [37]. It contains many non-essential genes where foreign genes can be inserted without having any effect on *in vitro* and/or *in vivo* replication potential of the virus [38,39,40], making it a technically appropriate vector for the expression of foreign antigens of other swine diseases. It has been reported that gI and gE genes are associated with virulence, whereas they are not critical for the immunogenicity of PRV. Deleting TK and gE/gI genes results in reduced virulence and attenuated PRV without affecting its immunogenicity [6,7,40]. Various gene-deleted PRV recombinants have been developed as vaccine vectors to express foreign genes [41,42,43]. Hence, PRV could be used for the development of promising live virus-vectored vaccine candidates.

In this study, the establishment of a fosmid-based genetic manipulation platform for the PRV TJ strain facilitates the robust generation of recombinant PRVs with gene deletions/insertions. Based on traditional methods such as homologous recombination, it has been reported to be very inefficient and labor-intensive to generate recombinant PRVs, due to a series of plaque purification to purify the recombinant viruses. Bacterial artificial chromosome (BAC) modification is a powerful tool for herpesviruses [44,45], but it takes several months and may result in genetic and phenotypic variations due to the presence of sequences for BAC vector [46,47]. On the contrary, fosmid library platform is a robust toolkit for the generation of recombinant viruses as described by recently published studies [48,49]. With this system, only the fragments from viral and/or bacterial genomic DNA are included, and seamless genetic modification is achieved without any alternation, in contrast to the fragments constructed by restriction enzymes. Consequently, for enhancing the efficiency and quality of genetic manipulation and rapid generation of recombinant PRVs we used the fosmid library platform. 

The US region of PRV has been demonstrated to be suitable for the insertion of various foreign genes, such as US4, US7, US8 and US9 [5,33,39,50]. Various studies have implicated that deleting genes leads to reduced replication potential of PRV and the virulence is associated directly with the capability of the virus to proliferate in the nasal epithelium [7]. Reduction in viral replication leads to lower antigen expression levels and reduced immunogenic capacity of the gene-deleted PRV mutants [51]. Typically, the ideal vaccine vector must be safe, and the inserted foreign genes should not restrict the immunogenicity or growth properties when compared with the parental virus [52]. Our previous study showed that rPRVTJ-delgE/gI-E2 and rPRVTJ-delgE/gI/TK-E2 were highly safe and immunogenic for pigs and capable of protecting the immunized pigs when challenged with a lethal PRV TJ strain [33,53]. These evidence suggest that rPRVTJ-delgE/gI/TK could be used as a biologically safe vector to express antigens from other swine pathogens. Considering the excellent safety and efficacy, we inserted the genes encoding the E2 of CSFV and Cap of PCV2 into the genome of PRVTJ to demonstrate its potential in vaccine development aiming to produce a novel multivalent vaccine candidate against PR, CSF and PCVAD. 

We partially deleted TK and gE/gI genes, keeping a balance between the immunogenicity and virulence of the recombinant virus. We inserted the E2 expression cassette after US9 and Cap was fused with gG (US4) using a fosmid based genetic manipulation platform, which was mediated by Red/ET recombination, resulting in seamless modification of the PRV genome. The recombinant virus was efficiently rescued, and it was free of any genetic alteration. Our data suggest that the replication and growth kinetics of rPRVTJ-delgE/gI/TK-E2-Cap were comparable to that of parental virus rPRVTJ-delgE/gI/TK *in vitro* and the insertion of foreign genes had no effect on the genetic stability of rPRVTJ-delgE/gI/TK-E2-Cap. Rabbits and pigs immunized with rPRVTJ-delgE/gI/TK-E2-Cap elicited strong PRV-specific antibody responses and did not exhibit any PRV-specific clinical signs. However, there were no detectable antibodies against the E2 and Cap in the immunized rabbits or pigs, possibly due to the unbefitting sites for the E2 and Cap insertion or because the expressed antigens were not able to stimulate the immune response to develop neutralizing antibodies. E2 of CSFV is an ideal antigen used for developing recombinant CSF vaccines, like DNA vaccines [54], subunit vaccines [55], and viral-vectored vaccines [53]. Nevertheless, some of these vaccine candidates were not able to fully protect from the virus [56,57], or before challenge there were no detectable neutralizing antibodies in the vaccinated pigs [54]. Various studies have shown that the ability of heterologous proteins to produce humoral antibodies might be related to various forms of the protein expression, such as secreted proteins, and the proteins expressed intracellularly or on the surface of the cells [58]. Perhaps it is the form of heterologous protein expression that affects the localization and antigen concentration to efficiently stimulate the immune system or the effectiveness of an antigen representation by the major histocompatibility complex (MHC) class II [59,60].

In summary, we report here the construction of a recombinant virus rPRVTJ-delgE/gI/TK-E2-Cap based on the fosmid library platform. The recombinant virus expressed E2 protein of CSFV and Cap protein of PCV2 *in vitro* was genetically stable, and the deleted/inserted genes had no effect on the phenotype of the recombinant virus. Upon inoculation in rabbits and pigs, no clinical signs were observed, and PRV-specific neutralizing antibodies were detected, however, there were no detectable antibodies against E2 and Cap. Therefore, further research is desirable to optimize the design and construction of rPRVTJ-delgE/gI/TK-E2-Cap which could be used as a vaccine candidate.

## 4. Materials and Methods 

### 4.1. Virus and Cells

The PRV TJ strain isolated from a swine herd from Tianjin, China in 2011 was used in this study, (GenBank accession number: KJ789182.1), and preserved at *−*80°C and amplified in the porcine kidney 15 (PK-15) cell line [61]. Vero and PK-15 cells were procured from China Center for Type Culture Collection (CCTCC, Wuhan, China) and cultured in Dulbecco’s modified Eagle’s medium (DMEM) (Thermo-Fisher Scientific, Carlsbad, CA, USA) supplemented with 10% heat-inactivated fetal bovine serum (FBS), plus 1% Antibiotic-Antimycotic (10,000 I.U./mL of penicillin, 10,000 µg/mL of streptomycin, and 25 µg/mL of amphotericin B. Gibco™, Grand Island, NY, USA) and incubated at 37°C with 5% CO_2_.

### 4.2. Construction of Recombinant Fosmids with TK and gE/gI Gene-Deletion

**Figure 5:**
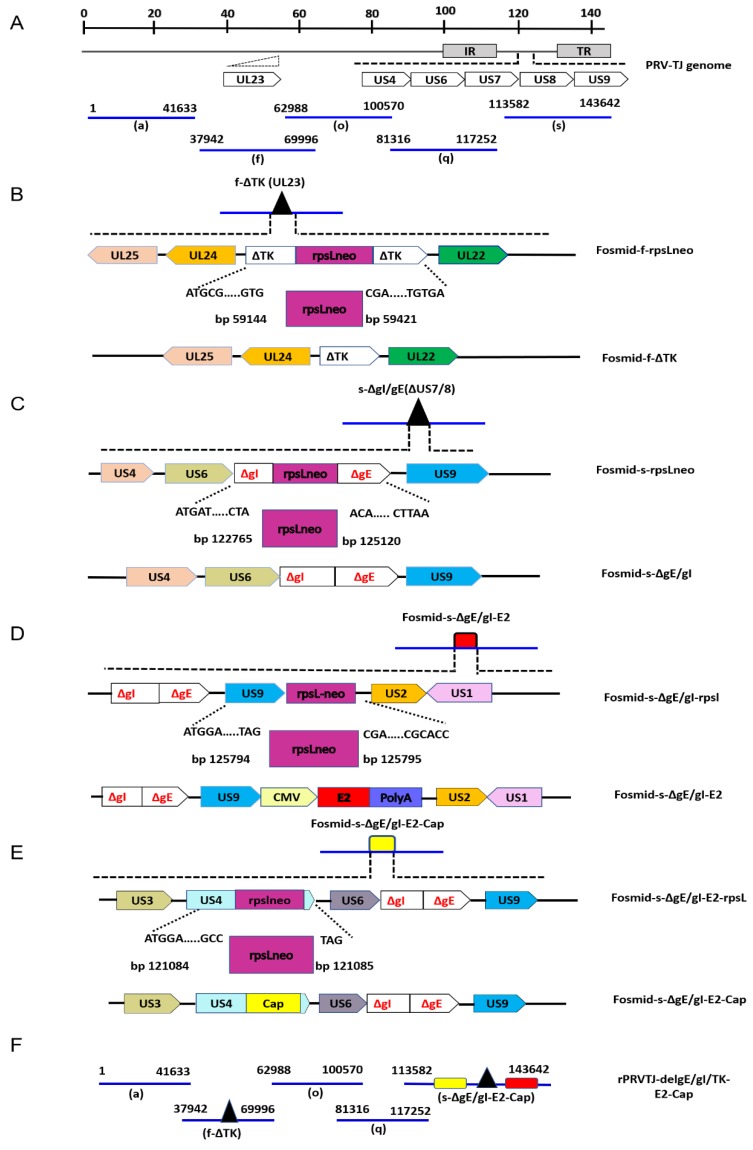
Construction of recombinant fosmids with TK/gE/gI deletions, and E2 and Cap gene insertions. **A.** The genomic organization of PRV, and the five fosmids generated from the PRV-TJ strain used for virus rescue. Numbers representing the fosmids names and the position of each fosmid fragment in the genome of PRV-TJ strain. **B.** Schematic diagram representing modification procedure for the intermediate fosmid and Fosmid-f-ΔTK. For fosmid modification, the rpsL-neo counter-selection marker cassette (rpsL-neo) harboring 50 bp left and right oligonucleotide homologous arms was inserted between 59144 and 59421 bp of PRV-TJ genome by the Red/ET-mediated recombination. Partially deleted TK (ΔTK) gene fragment with flanking homologous arms was used for replacing the rpsL-neo counter-selection marker to construct Fosmid-f-ΔTK. **C.** Schematic diagram representing modification procedure of the intermediate fosmid and Fosmid-s-ΔgE/gI. Partially deleted gE/gI (ΔgE/gI) genes fragment with 50 bp left and right oligonucleotide homologous arms was inserted between 122765 and 125120 bp of PRV-TJ genome as described above to generate Fosmid-f-ΔgE/gI. **D.** The schematic diagram representing modification procedure of the intermediate fosmid and the Fosmid-s-ΔgE/gI-US9E2. The E2 expression cassette harboring 50 bp left and right oligonucleotide homologous arms inserted between 125794 and 125795 bp (US9) of PRV-TJ genome. **E.** The schematic diagram representing modification procedure of the intermediate fosmid and Fosmid-s-ΔgE/gI-US9E2-US4Cap. The Cap gene flanked by 50 bp left and right oligonucleotide homologous arms was fused between the last amino acid and the stop codon of gG (US) i.e., 121084 and 121085 bp of the PRVTJ genome. **F.** Fosmid-f-ΔTK, s-ΔgE/gI-US9E2-US4Cap and other fosmids used to rescue rPRVTJ-delgE/gI/TK-E2-Cap.

The fosmid library platform for the PRV TJ strain established by Zhou et al., 2018 [62] was used in this study for the construction of rPRVTJ-delgE/gI/TK-E2-Cap (Figure 5A). Deleting TK and gE/gI genes resulted in reduced virulence and attenuated PRV without affecting its immunogenicity [6,7,40]. The gE/gI/TK-gene-deficient fosmids were constructed for the establishment of the vaccine platform to rationally attenuate the PRV TJ strain, and used as a vector for vaccine development. The detailed procedure for deleting these genes is summarized in Figure 5B,C. The gE, gI and TK genes were partially deleted in fosmids using a Counter Selection BAC Modification Kit (Gene Bridges, Berkeley, CA, United States) following the manufacturer’s protocol. In brief, firstly the desired fosmid and the plasmid for Red/ET expression (pRed/ET) and were co-electroporated into competent cells (E. coli DH10B). Subsequently, using specific primers, the rpsL-neo (counter-selection antibiotic marker) with flanking homologous arms was amplified by PCR (Table 3) and inserted at the desired position of the desired fosmids by the Red/ET-mediated recombination. Finally, the linear DNA fragments (partially deleted TK and gE/gI gene amplified by PCR) with specific homologous arms were electroporated into competent cells for replacing the rpsL-neo counter-selection antibiotic maker by Red/ET-mediated recombination.

### 4.3. Construction of Recombinant E2 Expression Cassette

The expression cassette for CSFV E2 was designed by overlapping PCR based on a plasmid pOK-12 backbone to design the recombinant transfer vector. The CSFV E2 was amplified from the CSFV Shimen strain and human cytomegalovirus (hCMV) promoter and polyA were amplified from the plasmid pEGFP-N1 (Clontech, Mountain View, CA, USA) [33]. To construct the transfer vector pOK12-CMV-E2-PolyA, the CMV, E2 and polyA were amplified by overlapping PCR, having the *Eco*RI and *Xba*I restriction sites using the purified fragments of CMV, E2, and polyA as the templates with specific primers and ligated into the *Xba*I and *Eco*RI sites of pOK-12. The resultant transfer plasmid pOK12-CMV-E2-polyA was analyzed using restriction enzyme digestion, sequencing, immunofluorescence and Western blotting assays.

### 4.4. Construction of Recombinant Fosmid with E2 and Cap Gene Insertion

The US region of PRV has proven to be suitable for the insertion of various foreign genes, such as US4, US7, US8 and US9 [5,33,39,50]. Therefore, in this study we selected some new target sites of the US region to insert foreign genes. The E2 expression cassette was inserted after US9 region and Cap was fused with gG (US4) of PRV TJ strain. The detailed procedure for inserting E2 and Cap is summarized in Figure 5D,E. Briefly, the linear DNA fragment of E2 expression cassette harboring 50bp left and right homology arms was amplified and inserted after the US9 region of fosmid-s-ΔgE/gI by Red/ET mediated recombination using the Counter Selection BAC Modification Kit as described earlier. The PCV2 Cap was codon-optimized and cloned into plasmid pUC57 (Genscript, USA). Then the Cap gene flanked by 50 bp left and right homologous arms (for US4) was amplified with specific primers **(**Table 3**)** from plasmid pUC57-Cap. The Cap gene was fused between the last amino acid and the stop codon of US4 region (gG) into the fosmid-s-ΔgE/gI-E2 by the Red/ET-mediated recombination as described previously. The modified fosmid (fosmid-s-ΔgE/gI-E2-Cap) carrying the E2 and Cap genes was confirmed using PCR and sequencing.

Finally, the recombinant virus was rescued by transfection into Vero cells with a group of five overlapping fosmids combinations (with or without TK/gE/gI deletion and E2/Cap insertion) covering the entire genome of the PRV TJ strain (Figure 5F).

### 4.5. Rescue and Dentification of rPRVTJ-delgE/gI/TK-E2-Cap

The resultant fosmids, fosmid-f-ΔTK, fosmid-s-ΔgE/gI, fosmid-s-ΔgE/gI-US9E2 and fosmid-s-ΔgE/gI-US9E2-US4Cap were extracted with a QIAfilter plasmid midi kit (Cat. 12243, Qiagen, Germany) and the concentration (ng/µL) was determined by NanoDrop^TM^ 2000 (Thermo-Fisher Scientific; Table 4). The recombinant virus was rescued as follows: a set of five fosmids combinations (with or without gE/gI/ TK deletion and E2/Cap insertion) covering the entire genome of the PRV TJ strain were used for virus rescue. Before transfection, the purified fosmids were digested with *Ase*I to release viral DNA inserts. Each fosmid DNA was adjusted to a concentration of 2µg for transfection into Vero cells using the X-treme GENE HP DNA transfection reagent (Roche, Germany). Vero cells transfected with five un-modified fosmids and one missing fosmid served as positive and negative controls, respectively (Table 4). At 72-96 hpt, the typical PRV CPE were observed on Vero cells. The supernatant from CPE-positive Vero cells was collected and filtered through syringe filter (pore size: 0.22 µm) and used for infecting PK-15 cells for further characterization of the recombinant virus. To confirm that the E2 and Cap genes were accurately inserted into at the desired position, viral genomic DNA was isolated and verified by PCR analysis with the specific primers matching TK, gE/gI, US9 and US4 sites (Table 3). The PCR products were purified and verified by sequencing. The recombinant virus was further identified by electron microscopy. Briefly, PK-15 cells were infected with rPRVTJ-delgE/gI/TK-E2-Cap, rPRVTJ-delgE/gI/TK and PRVTJ. The cell culture medium was harvested 36 h post-infection (hpi). The sample was centrifuged for 10 min at 3000× *g* and the supernatant was collected which was then subjected to again centrifugation for 10 min at 10,000× *g*, and pellet was resuspended in PBS. The virus samples were negatively stained with 2% phosphotungstic acid, the morphology of the recombinant viruses and PRVTJ particles were observed using an electron microscope. 

### 4.6. Immunofluorescence Assay (IFA) and Western Blotting

E2 and Cap proteins expression by rPRVTJ-delgE/gI/TK-E2-Cap was confirmed by IFA and Western blotting, as described previously [53]. The tissue culture infective dose (TCID_50_) of rPRVTJ-delgE/gI/TK-E2-Cap (TCID_50_) was 10^7^. For IFA, PK-15 cells cultured in 96-well plate were infected with rPRVTJ-delgE/gI/TK-E2-Cap at an MOI of 1 and incubated at 37°C in a CO_2_ incubator for 48 h. To fix the cells, 200 µL of chilled absolute ethanol was added to each well and placed at -20°C for 20 min. The cells were then air-dried and incubated with 100 µL (1:300 in PBS) of anti-E2 monoclonal antibody (MAb) HQ06 [63] and anti-Cap MAb 36A9 (Ingenasa, Madrid, Spain) [64] for 2 h at 37°C, subsequently washed thrice with phosphate-buffered saline (PBS), and incubated with fluorescein isothiocyanate (FITC)-conjugated anti-mouse IgG (Sigma-Aldrich, USA) for 1 h at 37°C. After washing the cells three times with PBS, the plates were visualized under a fluorescence microscope (Nikon TE200; Japan). The expression of the E2 and Cap proteins was further determined by Western blotting in CSFV, rPRVTJ-delgE/gI/TK-E2-Cap, rPRVTJ-delgE/gI/TK-Cap and rPRVTJ-delgE/gI/TK-infected PK-15 cells in 6-well plates by previously described procedure. The cells were lysed by treatment with lysis buffer (10 mM Tris-HCl, pH 7.4, 1 mM MgCl_2_ 0.5% NP-40, 20 µg/mL DNase I) and phenylmethylsulfonyl fluoride (PMSF) protease inhibitor. The cell lysate was cleared by centrifugation at 12,000× *g* for 5 min and the supernatant was collected. Separation of proteins was performed by 10% sodium dodecyl sulfate-polyacrylamide gel electrophoresis (SDS-PAGE) and afterwards transferred onto nitrocellulose membranes (Bio-Rad, USA). The membranes were then incubated with the anti-E2 MAb HQ06 and anti-Cap MAb 36A9 (1:300) at room temperature for 2 h, washed thrice by PBS and incubated with IRDye 800CW-labeled anti-mouse secondary antibody (Li-Cor) for 1 h. The lysate of non-infected PK-15 cells served as mock. The E2 and Cap proteins bands were visualized using the Odyssey infrared imaging system, and representative images were captured.

### 4.7. Growth Properties and Genetic Stability of the Recombinant Virus

To analyze the growth properties of recombinant viruses, PK-15 cells cultured in 24-well plates were infected with rPRVTJ-delgE/gI/TK-E2-Cap, rPRVTJ-delgE/gI/TK or PRV TJ at an MOI of 10 and placed on ice for 1 h. Subsequently, the cells were washed with pre-warmed fresh DMEM and incubated at 37°C for 1 h and rinsed by citrate buffer (pH 3.0) for 2 min for inactivating unabsorbed virus, if any. Then 500 µL of fresh DMEM was added to each well and cells were incubated at 37°C with 5% CO_2_. The cultures were harvested at 0, 4, 8, 12, 16, 20, 24, 28, 36, 48, 60, and 72 hpi. The titers of all the samples collected at various time-points were determined in duplicates in PK-15 cells, and the average of each was calculated as described previously [61]. Similarly, rPRVTJ-delgE/gI/TK-E2-Cap, rPRVTJ-delgE/gI/TK and PRVTJ were serially diluted 10-fold in DMEM and 100 µL of each sample was inoculated onto monolayers of PK-15 cell in 6-well plates. After incubation at 37°C for 1 h, the cells were washed two times with DMEM and overlaid by 2 mL of DMEM containing 1% low melting point agarose. The plaque-forming units (PFUs) were determined at 5 dpi [62]. To evaluate the genetic stability, the recombinant rPRVTJ-delgE/gI/TK-E2-Cap was passaged 20 times in PK-15 cells and the deleted TK and gE/gI genes, and the inserted E2 and Cap genes were verified using PCR and sequencing after every 5^th^ passage (F5,F10,F15,F20). Similarly, the expression of E2 and Cap was confirmed after every 5^th^ passage (F5,F10,F15,F20) by IFA using E2 and Cap-specific MAbs.

### 4.8. Safety and Immunogenicity Evaluation in Rabbits and Pigs

The animal experiments were conducted according to the Guide for the Care and Use of Laboratory Animals of Harbin Veterinary Research Institute (HVRI), Chinese Academy of Agricultural Sciences (CAAS), China. The animal experiment was approved by the Committee on the Ethics of Animal Experiments of HVRI, CAAS China. The ethics approval numbers are: HVRI-IACUC-2019-006 and HVRI-IACUC-2019-022 for rabbits and pigs, respectively. Thirty-five six-week-old rabbits confirmed free of CSFV, PRV and PCV2 by enzyme-linked immunosorbent assay (ELISA) and PCR were used to conduct the vaccine evaluation experiment. The rabbits were divided randomly into seven groups of five each (Table 5). Groups A to C were each injected intramuscularly (i.m.) with 1 mL of different doses (10^7^, 10^6^, and 10^5^ 50% tissue culture infective doses (TCID_50_) of rPRVTJ-delgE/gI/TK-E2-Cap; groups D and E were each inoculated i.m. with rPRVTJ-delgE/gI/TK (10^5^ TCID_50_) or 1 mL of DMEM, respectively; groups F and G, in a separate pen, were injected i.m. with one-dose of C-strain vaccine and one-dose of PCV2 vaccine, respectively. Three weeks after the first immunization, 10^7^, 10^6^ and 10^5^ TCID_50_ rPRVTJ-delgE/gI/TK-E2 groups (groups A-C), and 1 dose of C-strain vaccine one dose of PCV2 vaccine were boosted with the same vaccine and dose as those in the first immunization. Six weeks post-immunization various tissues samples, for example, heart, liver, lungs, kidneys and spleen, were collected from the rabbits and subjected to PCR and pathological examinations.

Similarly, fifteen six-week-old pigs were divided randomly into three groups of five each **(**Table 6**)**. All the pigs were checked and confirmed free from PRV, CSFV and PCV2 by ELISA and PCR. Group A was inoculated i.m. with 10^6^ TCID_50_ rPRVTJ-delgE/gI/TK-E2-Cap, group B was inoculated with 10^6^ TCID_50_ rPRVTJ-delgE/gI/TK, and group C was inoculated with 1mL of DMEM. Three weeks after the first immunization, pigs were boost-immunized with the same vaccine and dose as those in the first immunization. 

### 4.9. Blocking ELISA

Sera collected from rabbits and pigs were analyzed to detect gB-, gE-, E2- or Cap-specific antibodies by blocking ELISA using the PRV, CSFV antibody detection kits (IDEXX, USA) or PCV2 antibody detection kit (Ingenasa, Madrid, Spain) according to the manufacturer’s instructions. 

### 4.10. Statistical Analysis

Data analyses were performed using SPSS 14.0 software and GraphPad Prism, version 5 (Graph Pad Software, USA. The mean and standard deviation were calculated for antibody responses and statistical differences between various groups were analyzed by *t*-test (*P* < 0.05).

## Figures and Tables

**Table 1:** The anti-gB antibodies in the sera of the rabbits immunized with the viruses (S/N).

Groups Viruses		Days Post-Prime Immunization (Weeks Post-Booster Immunization)						
		0	7	14	21 (1)	28	35 (2)	42
A	rPRVTJ-delgE/gI/TK-E2-Cap 10^7^	≥0.8	0.66 ± 0.164 ^a^	0.489 ± 0.166	0.387 ± 0.129 ^a^	0.129 ± 0.023 ^a^	0.137 ± 0.041 ^a^	0.182 ± 0.056
B	rPRVTJ-delgE/gI/TK-E2-Cap 10^6^	≥0.8	0.94 ± 0.226	0.543 ± 0.248	0.429 ± 0.155	0.259 ± 0.016 ^b^	0.342 ± 0.156 ^b^	0.296 ± 0.135
C	rPRVTJ-delgE/gI/TK-E2-Cap 10^5^	≥0.8	0.991 ± 0.212	0.591 ± 0.256	0.514 ± 0.132	0.358 ± 0.162	0.405 ± 0.178	0.348 ± .0264
D	rPRVTJ-delgE/gI/TK 10^5^	≥0.8	0.408 ± 0.07	0.296 ± 0.042	0.194 ± 0.104	0.086 ± 0.015	0.13 ± 0.041	0.218 ± 0.076
E	DMEM	≥0.8	1.011 ± 0.061	1.031 ± 0.036	0.948 ± 0.029	0.93 ± 0.04	1.107 ± 0.079	1.0128 ± 0.03
F	C-strain Vaccine	—	—	—	—	—	—	—
G	PCV2 Vaccine	—	—	—	—	—	—	—

^a^ significant difference between the rPRVTJ-delgE/gI/TK-E2-cap (10^7^ or 10^5^ TCID_50_) (*P* < 0.05). ^b^ significant difference between the rPRVTJ-delgE/gI/TK-E2-cap (10^7^ or 10^6^ TCID_50_) (*P* < 0.05) “—” not determined.

**Table 2:** The anti-gB antibodies in the sera of the pigs immunized with the viruses (S/N).

Groups Viruses		Days Post-Prime Immunization (Weeks Post-Booster Immunization)						
		0	7	14	21 (1)	28	35 (2)	42
A	rPRVTJ-delgE/gI/TK-E2-Cap 10^6^	≥0.8	0.493 ± 0.132	0.374 ± 0.088	0.481 ± 0.098	0.064 ± 0.016	0.05 ± 0.009	0.06 ± 0.017
B	rPRVTJ-delgE/gI/TK 10^6^	≥0.8	0.424 ± 0.08	0.346 ± 0.1	0.455 ± 0.278	0.061 ± 0.016	0.073 ± 0.016	0.073 ± 0.014
C	DMEM	≥0.8	0.846 ± 0.061	0.865 ± 0.024	0.897 ± 0.033	0.854 ± 0.036	0.943 ± 0.056	0.885 ± 0.025

Statistically no significant difference between the groups A and B.

**Table 3:** Sequences of oligonucleotides used for PCR in this study.

Names	Sequences (5′-3′)	Target Gene
delTK-rpsL-F	CGTGATCTCCTCGCCGCCCGGGGGCACGGCGGCGGCGAGGAGGCGCGCCG GGCCTGGTGATGATGGCGGGATCG	rpsL
delTK-rpsL-R	CGGCGCGCGCCCCCAGTCGTCGCGCCAGCGGCGCCCCGAGCTCAGGTAGC TCAGAAGAACTCGTCAAGAAGGCG	rpsL
delTK-deleted-F	CGTGATCTCCTCGCCGCCCGGGGGCACGGCGGCGGCGAGGAGGCGCGCCG AGTCGCGCAGCTGGCACAGC	TK
delTK-deleted-R	CGGCGCGCGCCCCCAGTCGTCGCGCCAGCGGCGCCCCGAGCTCAGGTAGC GCGACGTGTTGACCAGCATG	TK
delTK-identify-F	CCGCGATCGCGATCACCGC	TK
delTK-identify-R	GCCCACGCGTGCACCTCGAG	TK
delgE/gI-rpsL-F	CGCCTGAGGGGGGCGAAGGGGTATCGCCTCCTGGGCGGTCCCGCGGACGC GGCCTGGTGATGATGGCGGGATCG	rpsL
delgE/gI-rpsL-R	TCGGCGGCCGGGTTCGAGACGCTCGTCGGGACGGGGGCGCTGGGGTCAAA TCAGAAGAACTCGTCAAGAAGGCG	rpsL
delgE/gI-deleted-F	CGCCTGAGGGGGGCGAAGGGGTATCGCCTCCTGGGCGGTCCCGCGGACGC CGACGAGCTAAAAGCGCAGC	gE/gI
delgE/gI-deleted-R	TCGGCGGCCGGGTTCGAGACGCTCGTCGGGACGGGGGCGCTGGGGTCAAA CGTGTCCATGTCGACGGAGG	gE/gI
delgE/gI-identify-F	GTGATCGTCGGCACGGGCAC	gE/gI
delgE/gI-identify-R	CTGCCGGCGTCCCACGCGG	gE/gI
US9-rpsL-F	TCTGCTCGCTGTCCGCGCTACTCGGGGGCATCGTCGCCAGGCACGTGTAG GGCCTGGTGATGATGGCGGGATCG	rpsL
US9-rpsL-R	GGGCGCGGCGGATGGGGGCGGGCCCCCGCTCCCGTTCGCTCGCTCGCTCG TCAGAAGAACTCGTCAAGAAGGCG	rpsL
US9-E2-F	TCTGCTCGCTGTCCGCGCTACTCGGGGGCATCGTCGCCAGGCACGTGTAG TAGTTATTAATAGTAATCAATTACG	POK12-E2
US9-E2-R	GGGCGCGGCGGATGGGGGCGGGCCCCCGCTCCCGTTCGCTCGCTCGCTCG TTAACCAGCGGCGAGCTGTTCTG	POK12-E2
US9-identify-F	CGCGCTACTCGGGGGCATCGTCGC	US9
US9-identify-R	CCGCTCCCGTTCGCTCGCTCGCTC	US9
US4-rpsL-F	TCAGGGCCCGGGCCCGGAACGACGGCTACCGCCACGTGGCCTCCGCCTGA GGCCTGGTGATGATGGCGGGATCG	rpsL
US4-rpsL-R	CACCCGGTGAGAGAGAGGGGGGAATCGCGGGGGAGTCGGGCGGGGCCGGG TCAGAAGAACTCGTCAAGAAGGCG	rpsL
US4-Cap-F	TCATCAGGGCCCGGGCCCGGAACGACGGCTACCGCCACGTGGCCTCCGCC ACCTACCCCCGCCGGAGGTAC	pUC57-Cap
US4-Cap-R	CCGGTGAGAGAGAGGGGGGAATCGCGGGGGAGTCGGGCGGGGCCGGGTCA TGGGTTCAGTGGGGGGTCC	pUC57-Cap
US4-identfy-F	GAGACCACCAACACCACCACC	US4
US4-identify-R	GTATGGGAACCTGGGGCGCCG	US4

**Table 4:** Fosmids combinations used for virus rescue.

Groups	Fosmids	Conc. (ng/µL)	Vol. (µL)	Transfection Mixture
PRVTJ Group-4 (PC)	a	601	3.32	Fosmids = 19.32 µL Transfection reagent = 30 µL DMEM = 950.68 µL
	f	506	3.95	
	o	550	3.63	
	q	440	4.54	
	s	515	3.88	
PRVTJ Group-4 (NC)	a	601	3.32	Fosmids = 15.44 µL Transfection reagent = 30 µL DMEM = 954.56 µL
	f	506	3.95	
	o	550	3.63	
	q	440	4.54	
	-	---	---	
Group-4 (rPRVTJ-delgE/gI/TK- E2-Cap)	a	601	3.32	Fosmids = 19.32 µL Transfection reagent = 30 µL DMEM = 950.68 µL
	f-ΔTK	486	3.95	
	o	550	3.63	
	q	440	4.54	
	s-ΔgE/gI-E2-Cap	515	3.88	

**Table 5:** Experimental design for immunization of rabbits.

Groups	No. of Rabbits	Viruses	Dose	Route
A	5	rPRVTJ-delgE/gI/TK-E2-Cap	10^7^ TCID_50_	i.m.
B	5	rPRVTJ-delgE/gI/TK-E2-Cap	10^6^ TCID_50_	i.m.
C	5	rPRVTJ-delgE/gI/TK-E2-Cap	10^5^ TCID_50_	i.m.
D	5	rPRVTJ-delgE/gI/TK	10^5^ TCID_50_	i.m.
E	5	DMEM	1mL	i.m.
F	5	PCV2 Vaccine	1 dose	i.m.
G	5	CSFV C-Strain Vaccine	1 dose	i.m.

**Table 6:** Experimental design for immunization of pigs.

Groups	No. of Pigs	Viruses	Dose	Route
A	5	rPRVTJ-delgE/gI/TK-E2-Cap	10^6^ TCID_50_	i.m.
B	5	rPRVTJ-delgE/gI/TK	10^6^ TCID_50_	i.m.
C	5	DMEM	1 mL	i.m.
